# Detection of movement onset using EMG signals for upper-limb exoskeletons in reaching tasks

**DOI:** 10.1186/s12984-019-0512-1

**Published:** 2019-03-29

**Authors:** Emilio Trigili, Lorenzo Grazi, Simona Crea, Alessandro Accogli, Jacopo Carpaneto, Silvestro Micera, Nicola Vitiello, Alessandro Panarese

**Affiliations:** 1 The BioRobotics Institute, Scuola Superiore Sant’Anna, Pisa, Italy; 2 IRCCS Fondazione Don Carlo Gnocchi, Milan, Italy; 3 Bertarelli Foundation Chair in Translational NeuroEngineering, Center for Neuroprosthetics and Institute of Bioengineering, School of Engineering, École Polytechnique Federale de Lausanne, Lausanne, Switzerland

**Keywords:** Upper-limb exoskeleton, Electromyography, Human-robot interface, Onset detection

## Abstract

**Background:**

To assist people with disabilities, exoskeletons must be provided with human-robot interfaces and smart algorithms capable to identify the user’s movement intentions. Surface electromyographic (sEMG) signals could be suitable for this purpose, but their applicability in shared control schemes for real-time operation of assistive devices in daily-life activities is limited due to high inter-subject variability, which requires custom calibrations and training. Here, we developed a machine-learning-based algorithm for detecting the user’s motion intention based on electromyographic signals, and discussed its applicability for controlling an upper-limb exoskeleton for people with severe arm disabilities*.*

**Methods:**

Ten healthy participants, sitting in front of a screen while wearing the exoskeleton, were asked to perform several reaching movements toward three LEDs, presented in a random order. EMG signals from seven upper-limb muscles were recorded. Data were analyzed offline and used to develop an algorithm that identifies the onset of the movement across two different events: moving from a resting position toward the LED (*Go-forward*), and going back to resting position (*Go*-*backward*). A set of subject-independent time-domain EMG features was selected according to information theory and their probability distributions corresponding to rest and movement phases were modeled by means of a two-component Gaussian Mixture Model (GMM). The detection of movement onset by two types of detectors was tested: the first type based on features extracted from single muscles, whereas the second from multiple muscles. Their performances in terms of sensitivity, specificity and latency were assessed for the two events with a leave one-subject out test method.

**Results:**

The onset of movement was detected with a maximum sensitivity of 89.3% for *Go-forward* and 60.9% for *Go-backward* events. Best performances in terms of specificity were 96.2 and 94.3% respectively. For both events the algorithm was able to detect the onset before the actual movement, while computational load was compatible with real-time applications.

**Conclusions:**

The detection performances and the low computational load make the proposed algorithm promising for the control of upper-limb exoskeletons in real-time applications. Fast initial calibration makes it also suitable for helping people with severe arm disabilities in performing assisted functional tasks.

## Background

Exoskeletons are wearable robots exhibiting a close physical and cognitive interaction with the human users. Over the last years, several exoskeletons have been developed for different purposes, such as augmenting human strength [1], rehabilitating neurologically impaired individuals [2] or assisting people affected by many neuro-musculoskeletal disorders in activities of daily life [3]. For all these applications, the design of cognitive Human-Robot Interfaces (cHRIs) is paramount [4]; indeed, understanding the users’ intention allows to control the device with the final goal to facilitate the execution of the intended movement. The flow of information from the human user to the robot control unit is particularly crucial when exoskeletons are used to assist people with compromised movement capabilities (e.g. post-stroke or spinal-cord-injured people), by amplifying their movements with the goal to restore functions.

In recent years, different approaches have been pursued to design cHRIs, based on invasive and non-invasive approaches. Implantable electrodes, placed directly into the brain or other electrically excitable tissues, record signals directly from the peripheral or central nervous system or muscles, with high resolution and high precision [5]. Non-invasive approaches exploit different bio-signals: some examples are electroencephalography (EEG) [6], electrooculography (EOG) [7], and brain-machine interfaces (BMI) combining the two of them [8–10]. In addition, a well-consolidated non-invasive approach is based on surface electromyography (sEMG) [11], which has been successfully used for controlling robotic prostheses and exoskeletons due to their inherent intuitiveness and effectiveness [12–14]. Compared to EEG signals, sEMG signals are easy to be acquired and processed and provide effective information on the movement that the person is executing or about to start executing. Despite the above-mentioned advantages, the use of surface EMG signals still has several drawbacks, mainly related to their time-varying nature and the high inter-subject variability, due to differences in the activity level of the muscles and in their activation patterns [11, 15], which requires custom calibrations and specific training for each user [16]. For these reasons, notwithstanding the intuitiveness of EMG interfaces, it is still under discussion their efficacy and usability in shared human-machine control schemes for upper-limb exoskeletons. Furthermore, the need for significant signal processing can limit the use of EMG signals in on-line applications, for which fast detection is paramount. In this scenario, machine learning methods have been employed to recognize the EMG onset in real time, using different classifiers such as Support Vector Machines, Linear Discriminant Analysis, Hidden Markov Models, Neural Networks, Fuzzy Logic and others [15–17]. In this process, a set of features is previously selected in time, frequency, or time-frequency domains [18]. Time-domain features extract information associated to signal amplitude in non-fatiguing contractions; when fatigue effects are predominant, frequency-domain features are more representative; finally, time-frequency domain features better elicit transient effects of muscular contractions. Before feeding the features into the classifier, dimensionality reduction is usually performed, to increase classification performances while reducing complexity [19]. The most common strategies for reduction are: i) feature projection, to map the set of features into a new set with reduced dimensionality (e.g., linear mapping through Principal Component Analysis); ii) feature selection, in which a subset of features is selected according to specific criteria, aimed at optimizing a chosen objective function. All the above-mentioned classification approaches ensure good performance under controlled laboratory conditions. Nevertheless, in order to be used effectively in real-life scenarios, smart algorithms must be developed, which are able to adapt to changes in the environmental conditions and intra-subject variability (e.g. changes of background noise level of the EMG signals), as well as to the inter-subject variability [20].

In this paper, we exploited a cHRI combining sEMG and an upper-limb robotic exoskeleton, to fast detect the users’ motion intention. We implemented offline an unsupervised machine-learning algorithm*,* using a set of subject-independent time-domain EMG features, selected according to information theory. The probability distributions of *rest* and *movement* phases of the set of features were modelled by means of a two-component Gaussian Mixture Model (GMM). The algorithm simulates an online application and implements a sequential method to adapt GMM parameters during the testing phase, in order to deal with changes of background noise levels during the experiment, or fluctuations in EMG peak amplitudes due to muscle adaptation or fatigue. Features were extracted from two different signal sources, namely onset detectors, which were tested offline and their performance in terms of sensitivity (or *true positive rate*), specificity (or *true negative rate*) and latency (*delay* on onset detection) were assessed for two different events, i.e. two transitions from rest to movement phases at different initial conditions. The two events were selected in order to replicate a possible application scenario of the proposed system. Based on the results we obtained, we discussed the applicability of the algorithm to the control of an upper-limb exoskeleton used as an assistive device for people with severe arm disabilities.

## Materials and methods

### Experimental setup

The experimental setup includes: (i) an upper-limb powered exoskeleton (NESM), (ii) a visual interface, and (iii) a commercial EMG recording system (TeleMyo 2400R, Noraxon Inc., AZ, US).

### NESM upper-limb exoskeleton

**Fig. 1:**
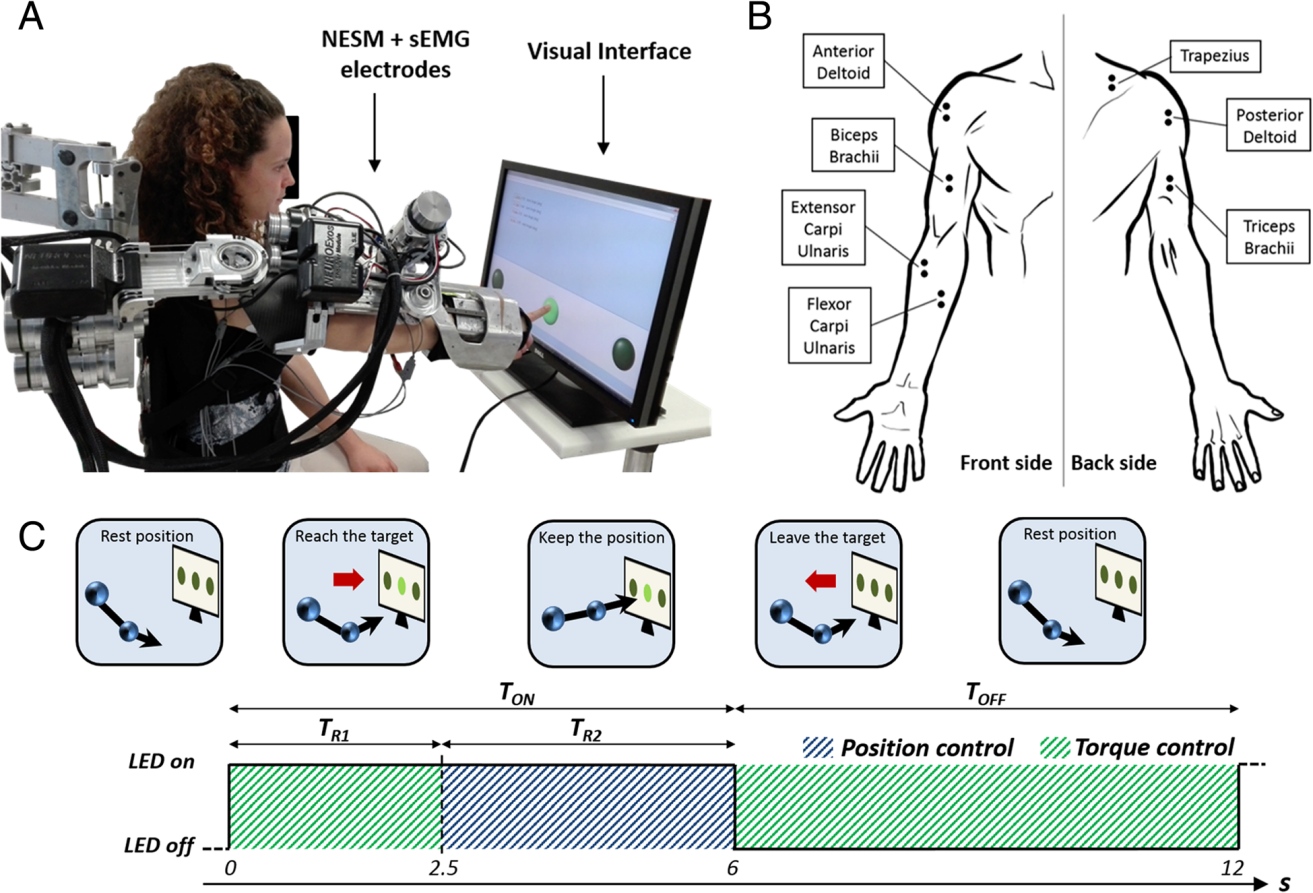
**a** Experimental setup, comprising NESM, EMG electrodes and the visual interface; **b** Location of the electrodes for EMG acquisition; **c** Timing and sequence of action performed by the user during a single trial

NESM (Fig. 1a) is a shoulder-elbow powered exoskeleton designed for the mobilization of the right upper limb [21, 22], developed at The BioRobotics Institute of Scuola Superiore Sant’Anna (Italy). The exoskeleton mechanical structure hangs from a standing structure and comprises four active and eight passive degrees of freedom (DOFs), along with different mechanisms for size regulations to improve comfort and wearability of the device.

The four active DOFs are all rotational joints and are mounted in a serial kinematic chain. Four actuation units, corresponding to the four active DOFs, allow the shoulder adduction/abduction (sAA), flexion/extension (sFE) and internal/external rotation (sIE), and the elbow flexion/extension (eFE). Each actuation unit is realized with a Series Elastic Actuation (SEA) architecture [23], employing a custom torsional spring [24] and two absolute encoders, to measure the joint angle and the joint torque as explained in [21]. SEAs allow reducing the mechanical stiffness of the actuator and easy implementation of position and torque controls.

The NESM control system runs on a real-time controller, namely a sbRIO-9632 (National Instruments, Austin, TX, US), endowed with a 400 MHz processor running a NI real-time operating system and a field programmable gate array (FPGA) processor Xilinx Spartan-3. The high-level layer runs at 100 Hz, whereas the low-level layer runs at 1 KHz.

### NESM control modes

#### Low-level control

The low-level layer allows the exoskeleton to be operated in two control modalities, namely *joint position* and *joint torque* control modes. In the position control mode, each actuator drives the joint position to follow a reference angle trajectory: this control mode is used if the arm of the user has no residual movement capabilities and needs to be passively guided by the exoskeleton. If the user has residual movement capabilities but is not able to entirely perform a certain motor task, the exoskeleton can be controlled in torque mode: each actuation unit can supply an assistive torque to help the user accomplish the movement; we refer to *transparent mode* when null torque is commanded as reference. Both control modes are implemented by means of closed-loop controllers, independent for each actuation unit. Controllers are proportional-integrative-derivative (PID) regulators, operating on the error between the desired control variable (angle or torque) and the measured control variable (joint angle or joint torque). Safety checks are implemented when switching from one control mode to the other, in order to avoid undesired movements of the exoskeleton.

#### High-level control

The high-level layer implements the control strategies to provide the movement assistance. A graphical user interface (GUI) has been implemented in LabVIEW environment. The GUI allowed to (i) set the desired control mode and control parameters, (ii) visualize joint angles, torques and EMG signals, (iii) launch the visual interface, and (iv) save data. NESM high-level controller also implements a gravity compensation algorithm to counteract the gravity torque due to the exoskeleton weight. A more detailed description of the control modes and their performances can be found in [21, 22].

### Visual interface

A visual interface (Fig. 1a) displayed three LEDs (west - W, center - C, and east - E) for the reaching movements, placed on different positions on a computer screen (15 cm apart, at left, center, and right, respectively). The visual interface was implemented in LabVIEW and launched by the NESM GUI.

### EMG recording and acquisition system

EMG signals from seven muscles of the right shoulder (Trapezius, Anterior and Posterior Deltoid), arm (Biceps and Triceps Brachii) and forearm (Flexor and Extensor Carpi Ulnaris) were amplified (1000x) and band pass-filtered (10–500 Hz) through a TeleMyo 2400R system (Noraxon Inc., AZ, US). The location of the electrodes is shown in Fig. 1b. The sbRIO-9632 interfaced the TeleMyo analog output channels: EMG signals were sampled by the FPGA layer at 1 kHz and sent to the real-time layer for visualization and data storage.

### Subjects

A total of 10 healthy subjects (8 male, 2 female, age 26 ± 5 years) participated in the experiment, and they all provided written informed consent. The procedures were approved by the Institutional Review Board at The BioRobotics Institute, Scuola Superiore Sant’Anna and complied with the principles of the declaration of Helsinki.

### Experimental protocol

Upon arrival, subjects were prepared for the experiment. Participants wore a t-shirt and were prepared for the application of the EMG electrodes over the skin according to the recommendations provided by SENIAM [25]. Then, subjects wore the exoskeleton with the help of the experimenter, and the size regulations were adjusted to fit the user’s anthropometry. The subjects sat in front of a screen showing the visual interface, having the center of the right shoulder aligned with the central LED, in order to allow symmetric movements toward left and right LEDs.

Seven sessions per subject were performed, each consisting of 24 reaching movements, with 5 min of rest between sessions to avoid muscular fatigue. The targets (i.e. the LEDs) were presented in random order. For each reaching trial, the subjects were instructed to:

- keep a resting position as long as all the LEDs were turned off,
- as soon as one LED turned on, move the arm towards it and touch the screen,
- keep the position (touching the screen) as long as the LED was turned on,
- as soon as the LED turned off, move back to the resting position.

Each trial was set to a duration of *T* = 12 *s*; within this duration, the LED was turned on for *T*_*ON*_ = 6 *s* (Fig. 1c). When the LED turned ON, the exoskeleton control mode was automatically set to transparent mode, to allow the subject to start the movement and reach the target. After *T*_*R*1_ = 2.5 *s* the control mode was automatically set to position control for a duration of *T*_*R*2_ = 3.5 *s*; notably *T*_*R*1_ was set long enough to ensure subjects could reach the target. When the LED turned OFF, subjects were asked to flex the elbow until the eFE measured torque exceeded the threshold *τ*_*thr*_ = 2 *N* ∙ *m*; this value was used to discriminate a voluntary action of the user, to switch again the exoskeleton control mode to transparent mode and let the subject move the arm back to the resting position. The LED was off for *T*_*OFF*_ = 6 *s* and then a new trial was started.

### EMG data processing and features extraction

The EMG signals were hardware-filtered on the Noraxon TeleMyo device with high-pass and anti-aliasing low-pass filters for all channels, to achieve a pass band between 10 and 500 Hz. Digital signals were then converted to analog by the Noraxon TeleMyo and sent to the analog-digital converter of the NESM FPGA layer, operating at a sampling frequency of 1 kHz. Although the cut-off frequency of the anti-aliasing filter was close to the theoretical Nyquist frequency, it was the best filtering options available with our hardware setup. For offline analysis, an additional high-pass filter (Butterworth, 4th order) with a cut-off frequency of 10 Hz was necessary to remove low-frequency components from data collected from the FPGA. Notch filter at 50 Hz was then used to eliminate residual powerline interference. We considered 14 time-domain features to extract information from the EMG signals [26]. Features were computed within a sliding window of 300 ms (10 ms update interval). A description of the features and their mathematical formulation can be found in the Appendix.

### Motion intention detection

For each trial, within each reaching movement, the EMG signals were segmented into two phases: *rest*, corresponding to the phase in which the upper limb was kept still in the initial resting position, and *movement,* corresponding to the phase in which the upper limb was moving towards or was voluntarily touching the target. This transition from *rest* to *movement* was defined as the *Go-forward* event.

**Fig. 2:**
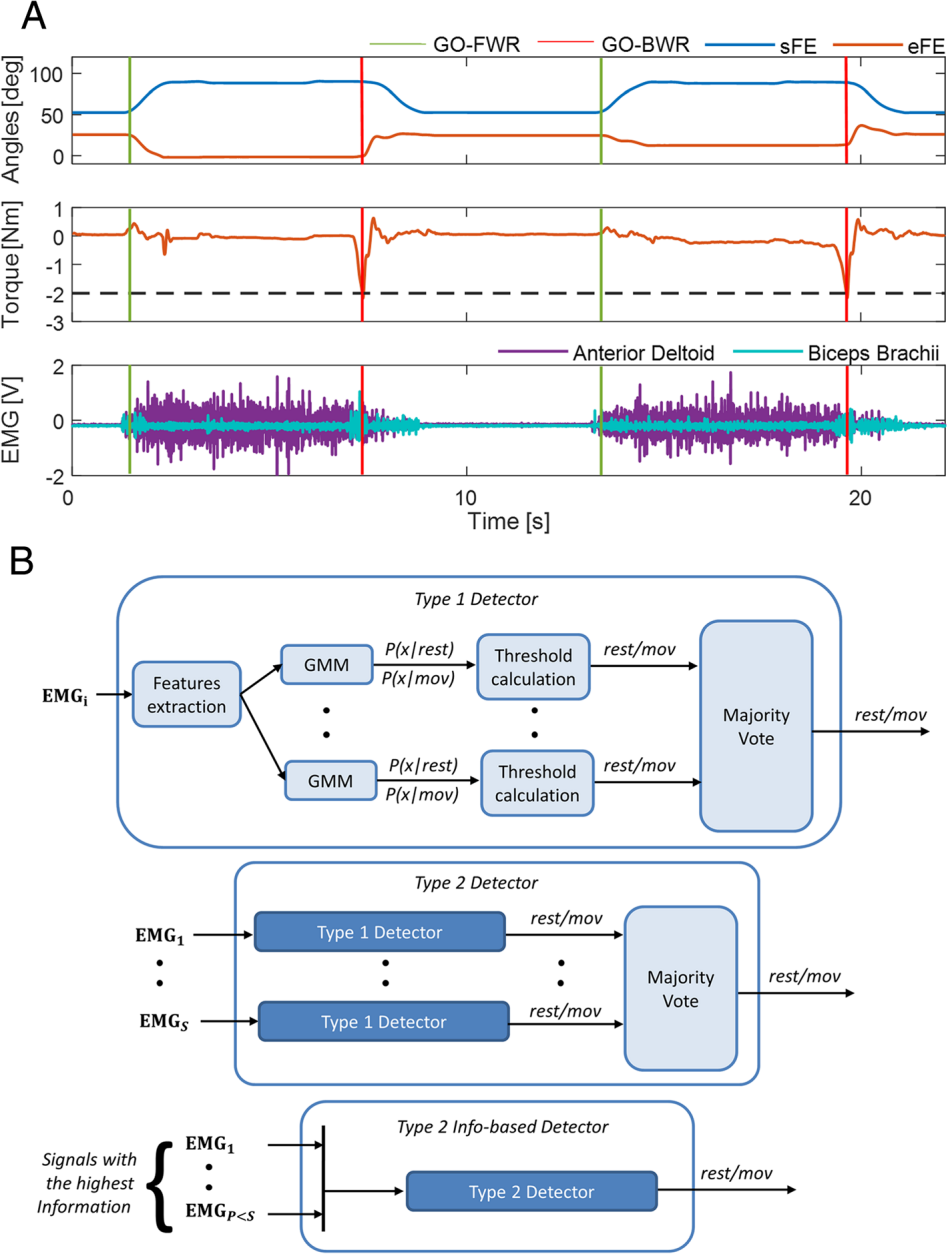
**a** Sample data acquired from one subject participating the experiments: joint angles from two representative joints of the upper-limb exoskeleton (for *Go-forward* discrimination); torque on the eFE joint for *Go-backward* discrimination; raw EMG data from two representative muscles. **b** Schematic representation of the detectors that were tested

A similar approach was adopted for retracting movements. The EMG signals were segmented into two phases: *rest*, corresponding to the phase in which the upper limb was held fixed near the target by the exoskeleton (in position control) and *movement*, corresponding to the phase in which the upper limb was moving (or trying to move, when the exoskeleton was in position control) to return to the initial resting position. The transition from *rest* to *movement* was defined as the *Go-backward* event. Figure 2a shows, for a representative subjects, kinematic and kinetic data used for the discrimination of the two events, together with the raw EMG signals for two representative muscles.

To detect both events, the probability distribution of each feature corresponding to *rest* and *movement* phases was modeled by a Gaussian Mixture Model (GMM), in which the density function of each feature is a linear mixture of two Gaussian curves, each representing the distribution of that feature within a given phase.

#### GMM training phase

The parameters of the two-components GMM were estimated using an unsupervised approach based on the Expectation Maximization (EM) algorithm [27]. The GMM probability density function is given by:

1 $$ p\left(x,{\lambda}_M\right)={w}_{rest}\bullet \frac{1}{\sqrt{2\pi {\sigma}_{rest}^2}}{e}^{-\frac{{\left(x-{\mu}_{rest}\right)}^2}{2{\sigma}_{rest}^2}}+{w}_{mov}\bullet \frac{1}{\sqrt{2\pi {\sigma}_{mov}^2}}{e}^{-\frac{{\left(x-{\mu}_{mov}\right)}^2}{2{\sigma}_{mov}^2}} $$

or, equivalently:

2 $$ p\left(x,{\lambda}_M\right)={w}_{rest}\bullet p\left(x| rest,{\lambda}_M\right)+{w}_{mov}\bullet p\left(x| mov,{\lambda}_M\right) $$

where *μ*_*rest*_ and *μ*_*mov*_ are the means, and ${\sigma}_{rest}^2$ and ${\sigma}_{mov}^2$ are the variances of the Gaussian distribution for the given phase *rest* and *movement*, respectively. The parameters *w*_*rest*_ and *w*_*mov*_ represent the a priori distribution of task in rest/movement phases. The modeling problem involves estimating the parameter set ${\lambda}_M=\left\{{w}_{rest},{w}_{mov},{\mu}_{rest},{\mu}_{mov},{\sigma}_{rest}^2,{\sigma}_{mov}^2\right\}$ from a training window of *M* ≤ *L* samples of the observed signal.

Given the training sequence *x*_1_, *x*_2_, … , *x*_*M*_, a Maximum Likelihood Estimation (MLE) of parameter set *λ*_*M*_ can be obtained by solving the problem:

3 $$ {\lambda}_M=\mathit{\arg}\underset{\lambda }{\mathit{\max}}\left[\ p\left(x,\lambda \right)\right] $$

which was tackled by iteratively applying the steps of the EM algorithm (1), until the difference between two consecutive estimations was lower than 10^−6^ for all parameters. The two estimated Gaussian distributions were then used to identify an optimal threshold, *θ*, that minimized the classification error:

4 $$ {w}_{rest}\bullet p\left(x=\theta | rest,{\lambda}_M\right)={w}_{mov}\bullet p\left(x=\theta | mov,{\lambda}_M\right) $$

The samples with feature value less than *θ* are classified as *rest*, while greater than *θ* as *movement*. At the end of the training, the parameters *λ*_*M*_ for each considered feature were obtained. These were employed as initial guesses for the parameters of the distributions sequentially estimated during the GMM testing.

For each subject, data of each session were used alternatively for the training phase and tested on data of the remaining 6 sessions. The outcome measures over all the testing sessions were then averaged.

#### GMM testing phase

Reiteration of the EM algorithm for each new sample acquired in the testing phase is disadvantageous in term of both computational load and consumption of memory; on the other hand, maintaining a fixed threshold during the testing phase could lead to inaccurate results due to changing background noise levels during the experiment, or varying EMG peak amplitudes as a result of muscle adaptation or fatigue.

Liu et al. [20] proposed a sequential method to adapt GMM parameters during the testing phase promoting computation efficiency. Here, the model is sequentially updated at each new observation *x*_*l* + 1_ every 10 ms, as follows:

5 $$ {w}_{i,l+1}=\alpha {w}_{i,l}+\left(1-\alpha \right)p\left(i|{x}_{l+1},{\lambda}_l\right) $$

6 $$ {\mu}_{i,l+1}=\frac{\alpha {w}_{i,l}{\mu}_{i,l}+\left(1-\alpha \right)p\left(i|{x}_{l+1},{\lambda}_l\right){x}_{l+1}}{w_{i,l+1}} $$

7 $$ {\sigma^2}_{i,l+1}=\frac{\alpha {w}_{i,l}{\sigma^2}_{i,l}+\left(1-\alpha \right)p\left(i|{x}_{l+1},{\lambda}_l\right){\left({x}_{l+1}-{\mu}_{i,l+1}\right)}^2}{w_{i,l+1}} $$

where *i* ∈ {*rest*, *mov*}, *λ*_*l*_ is the previous estimate of GMM parameters, and α indicates the forgetting factor *(*$\alpha =\frac{L-1}{L},0<\alpha \le 1$*)*. The conditional probability *p*(*i*| *x*_*l* + 1_, *λ*_*l*_) at the generic time instant *t* is given by:

8 $$ p\left(i|{x}_t,{\lambda}_l\right)=\frac{w_{i,l}p\left({x}_t|i,{\lambda}_l\right)}{w_{rest,l}p\left({x}_t| rest,{\lambda}_l\right)+{w}_{mov,l}p\left({x}_t| mov,{\lambda}_l\right)} $$

The new estimates of the GMM parameters, *λ*_*l* + 1_, can be derived from *λ*_*l*_ and *x*_*l* + 1_ using the above sequential scheme. Then, the time-varying threshold *θ*_*l* + 1_ can be determined from Equation (4) which decides whether *x*_*l* + 1_ is classified as *rest* or *movement*.

#### Subject-independent feature set

The selection of a subject-independent set of EMG features was performed by means of information theory tools. First, the information carried by each single feature about the *rest* and *movement* phases of movement was quantified for each recorded muscle. Then, the contributions to the computed information due to *Redundancy* and *Synergy* effects were assessed according to the information breakdown proposed by [28]. The *Redundancy* term takes into account the similarities in the distribution across phases of phase-conditional response probabilities of individual features, whereas the *Synergy* term quantifies the amount of information available from the feature-feature or movement phase-feature correlations.

In our study, two features are *synergic* if the information about the events carried when they are considered together is higher than the information conveyed by each feature alone. Similarly, features are *redundant* if they carry similar information about the events.

According to [28], the mutual information of the variables *F* (feature) and *R* (phase) can be written as the sum of four terms:

9 $$ I\left(\mathcal{R};\mathcal{F}\right)={I}_{lin}+{I}_{sig- sim}+{I}_{cor- ind}+{I}_{cor- dep} $$

where the *linear term*, *I*_*lin*_, quantifies the information obtained if each feature were to convey independent information on the movement phase; the *signal-similarity* term, *I*_*sig* − *sim*_, quantifies the *Redundancy* effects; and the correlation components, *I*_*cor* − *ind*_ + *I*_*cor* − *dep*_, quantify the *Synergy* effects.

Each contribution of the information breakdown was computed via the C- and Matlab-based Information Breakdown Toolbox (ibTB) developed by Magri et al. [29]. The selection of subject-independent features was carried on by assessing the *Redundancy* and *Synergy* terms of the information breakdown. The criteria for feature selection were: 1) to choose the features that minimize redundancy effects and 2) to maximize synergistic effects.

Information theory was also exploited to select the best window length for feature extraction, by comparing the information content of the features using 100, 300 and 500 ms. For *Go-forward*, information content of the features slightly decreased for increased window length. The differences were not significant when testing window length effect on the three samples (Friedman test; *p* > 0.05). However, when the samples were tested in pairs, information content at 500 ms was lower than both 300 and 100 ms (*p* < 0.05; Wilcoxon sign rank test). Instead, for *Go-backward*, information content did not change as the window length increased (both Friedman test and Wilcoxon sign rank test; p > 0.05). Based on these results, we selected 300 ms as the best choice for window length, in accordance to data found in literature about the optimal window length for the feature calculation. Indeed, several studies report a window length of 300 ms as the maximum limit allowed for feature extraction in online applications [18, 30]. Similar approaches exploiting information theory suggest an optimal window size between 200 and 300 ms [31].

#### Onset detector type

At each update of the observation window new EMG features were calculated, thus an equivalent number of classification outputs were available. We compared three types of detectors making decisions on whether the new output is *rest* or *movement* in different ways, and requiring different amounts of computational load and memory consumption (Fig. 2b):

1. **Type 1 detector:** it takes as input a number M of features computed on a single EMG signal; GMM algorithms work in parallel on each feature and the final decision is made by a majority voting procedure on their outputs: it is *rest* if the corresponding number of outputs are at least $\frac{M}{2}+1$, *movement* otherwise.
2. **Type 2 detector:** it takes as input the features computed on multiple EMG signals; each EMG signal is the input of a type 1 sub-detector, and the final decision is made by a majority voting on their outputs. A number S = 7 of EMG signals are used as input for type 2 detectors in this study. Additionally, a type 2 *info-based* detector has been tested, which takes as input the features computed on a subset of P < S EMG signals, i.e. the ones carrying the highest information. *P* = 3 has been chosen in order to have the minimum number of signal sources to make a majority voting

#### Performance metrics

Three parameters were used to evaluate the performances of the three types of detectors:

1. **Sensitivity** (or *true positive rate*): it measures the proportion of onset events that are correctly identified as such

11 $$ Sensitivity=\frac{TP}{TP+ FN} $$

2. **Specificity** (or *true negative rate*): it measures the proportion of correctly detected time samples not being classified as onset

12 $$ S\mathrm{p} ecificity=\frac{TN}{TN+ FP} $$

3. **Latency:** it measures the average delay of onset detection, with respect to the time instant of actual movement initiation, or reference onset time *t*_0_

13 $$ Latency={\left\langle {t}_d-{t}_0\right\rangle}_{trials} $$

where TP, TN, FN and FP are the number of true positives, true negatives, false negatives and false positives, respectively; *t*_*d*_ is the onset time detected by the algorithm and *t*_0_ is the reference onset time, i.e. the time instant on which the kinematic variables assumed a value corresponding to the 10% of their peak values during the movement (*Go-forward*) or the time instant on which the eFE measured torque overcomes the threshold value (*Go-backward*). The kinematic variables are the angular positions of the four active joints (sAA, sFE, sIE, eFE).

In addition, in order to assess the computational load, the algorithm has been implemented on a dual-core 667 MHz real-time processor (sbRIO-9651, National Instrument, US) and runs at 100 Hz sampling frequency. This processor has better performances with respect to the one used in the NESM, and will be employed in future versions of the exoskeleton. Raw sEMG are acquired at the FPGA level, running at 1 kHz, and sent by means of a direct memory access (DMA) method to the high-level control layer, for signal processing and feature extraction. With this FIFO-based method, during each iteration of the high-level control (i.e. 10 ms) ten sEMG samples (i.e. 1 ms data) are collected from the FPGA.

Data recorded during the experimental session, together with the initial GMM parameters obtained after the training phase, were used to run a simulation of the algorithm for 90 s (corresponding to 6 full cycles), and the maximum iteration duration of a single iteration was extracted.

## Results

### Movement onset analysis

#### Subject-independent set of EMG features

**Fig. 3:**
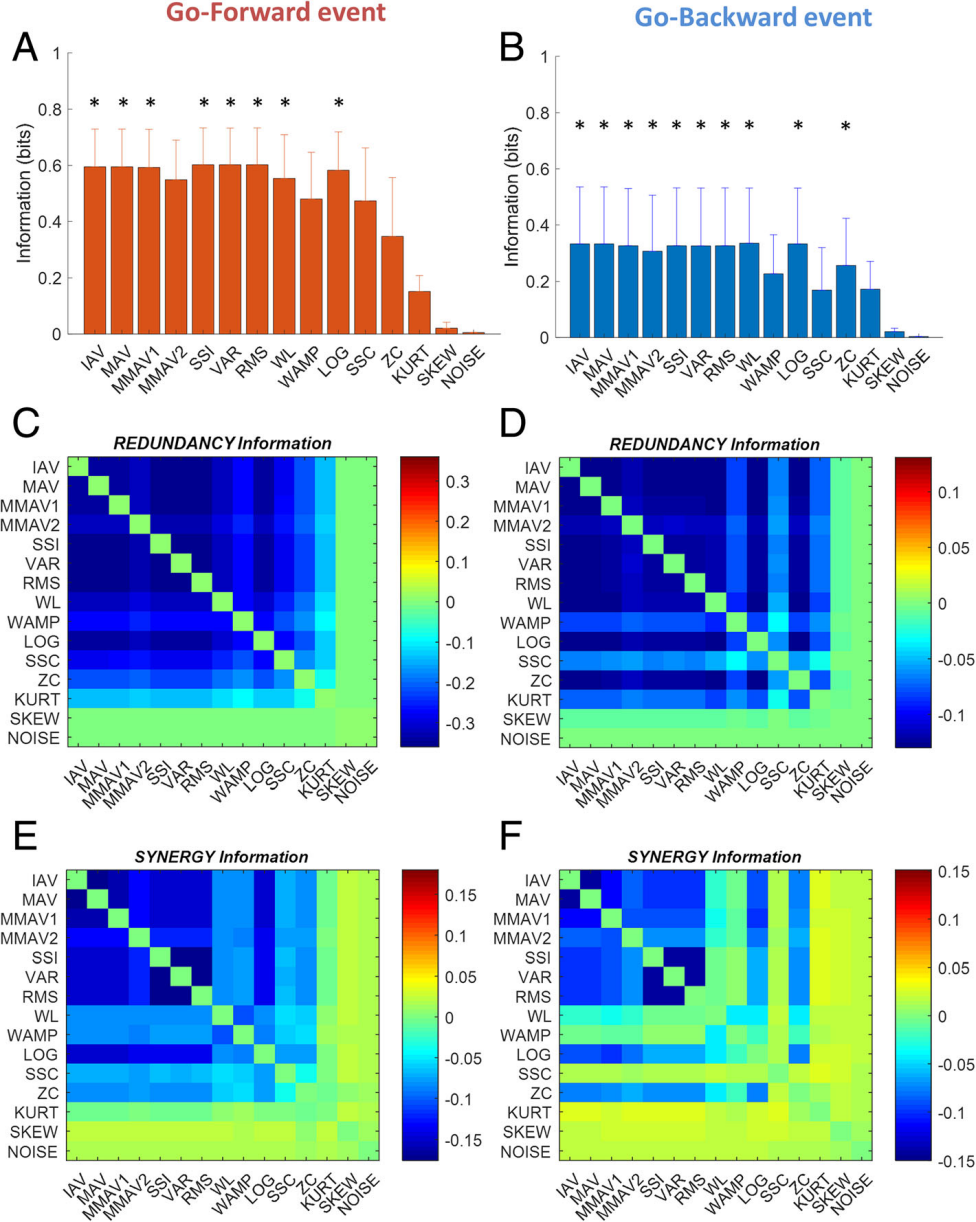
Information (in bit) carried by the fourteen selected features and white noise for *Go-forward* (**a**) and *Go-backward* (**b**). Colormap of the similarity term of the information breakdown for analysis of redundancy effects between features for *Go-forward* (**c**) and *Go-backward* (**d**). Colormap of the correlation term of the information breakdown for analysis of synergistic effects between features for *Go-forward* (**e**) and *Go-backward* (**f**)

Figure 3a and b show, for *Go-forward* and *Go-backward* onset detection respectively, the information about the rest/movement states carried by each of the 14 features (mean ± SD across subjects and muscles) and by white noise, the latter used as reference. Eight out of fourteen features (IAV, MAV, MMAV1, SSI, VAR, RMS, WL and LOG) carried significantly more information than white noise for both *Go-forward* and *Go-backward* (KW test; *p* < 0.001; Tuckey’s post hoc), and were selected for further in-depth analysis. Fig. 3c and d show, for each of the fourteen features in the two events, the similarity term (*I*_*sig* − *sim*_) of the information breakdown, thus the redundancy effect between pairs of features: the eight selected features all showed to be redundant to some degree, e.g. there were similarities in the distribution across rest/movement states of state-conditional response probabilities of individual features (2). Analogously, in Figure 3e and f, the correlation term (*I*_*cor* − *ind*_ + *I*_*cor* − *dep*_) is reported. Synergistic effects between features are expressed by positive correlation: IAV, MAV and MMAV1 features showed to be non synergistic (negative correlation) and similarly, RMS and SSI and VAR. For this reason we discarded MAV, MMAV1, RMS and VAR from the set of the eight most informative features, and considered the set {IAV, SSI, WL, LOG} as the most informative, most synergistic and less redundant subject-independent set of features to be used for EMG-based movement onset detection.

#### Information content of the extracted features

**Fig. 4:**
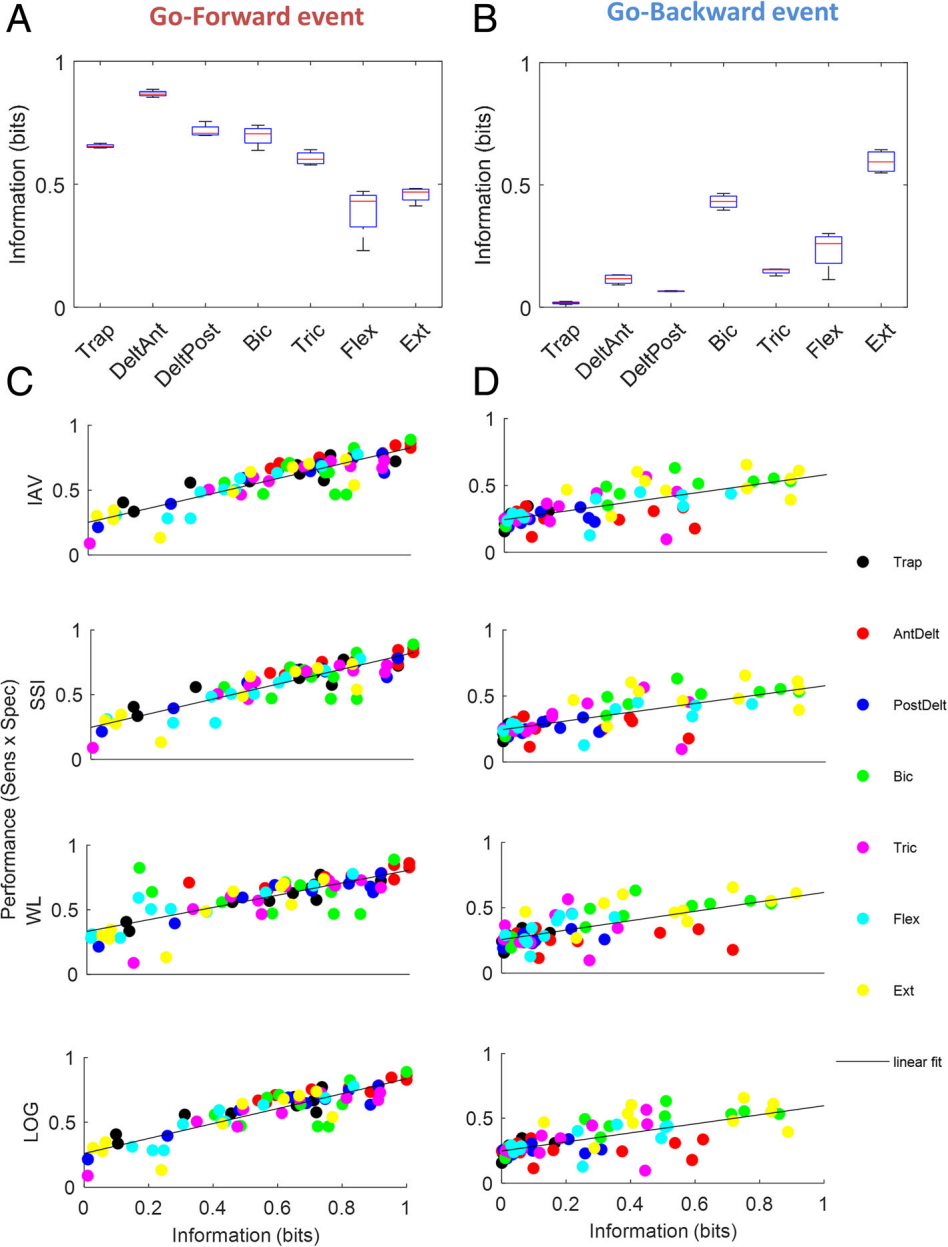
Information (in bit) carried by the 7 selected muscles considered as Type 1 Detectors for *Go-forward* (**a**) and *Go-backward* (**b**). Correlation between detector performances (sensitivity x specificity) and the information content of the four features selected for the testing phase in *Go-forward* (**c**) and *Go-backward* (**d**): 70 points are reported for each plot (10 subjects, 7 testing session for each)

After the optimal set of features had been selected, the information content of the 7 muscles has been calculated according to Eq. 9, in order to identify which muscles are more suitable as Type 1 detector for the two events. The results are reported in Figure 4a and b, for the two event respectively. Although for *Go-forward* all Type 1 detectors except Flexor and Extensor Carpi Ulnaris carry an information content higher than 0.5 bit (Anterior Deltoid being the most informative one with 0.87 bit of information), the same detectors result less informative for *Go-backward*, with the exception of Extensor Carpi Ulnaris (0.68 bit of information).

Figure 4c and d inspect the correlation between the performance of the detectors in terms of both sensitivity and specificity (i.e. their product), and the information content of the four selected features, calculated for each of the Type 1 detectors and for each subject. As reported in Table 1, all the features show statistically significant correlation for both events (Pearson correlation coefficient between 0.66 and 0.88, *p*-value< 0.001).

**Table 1:** Pearson correlation coefficients between sensitivity and information content of the five selected features

	Pearson correlation coefficient	
Features	*Go-Forward*	*Go-Backward*
*IAV*	0.87	0.70
*SSI*	0.87	0.71
*WL*	0.77	0.66
*LOG*	0.88	0.68

#### Performance of different EMG-based detectors

**Fig. 5:**
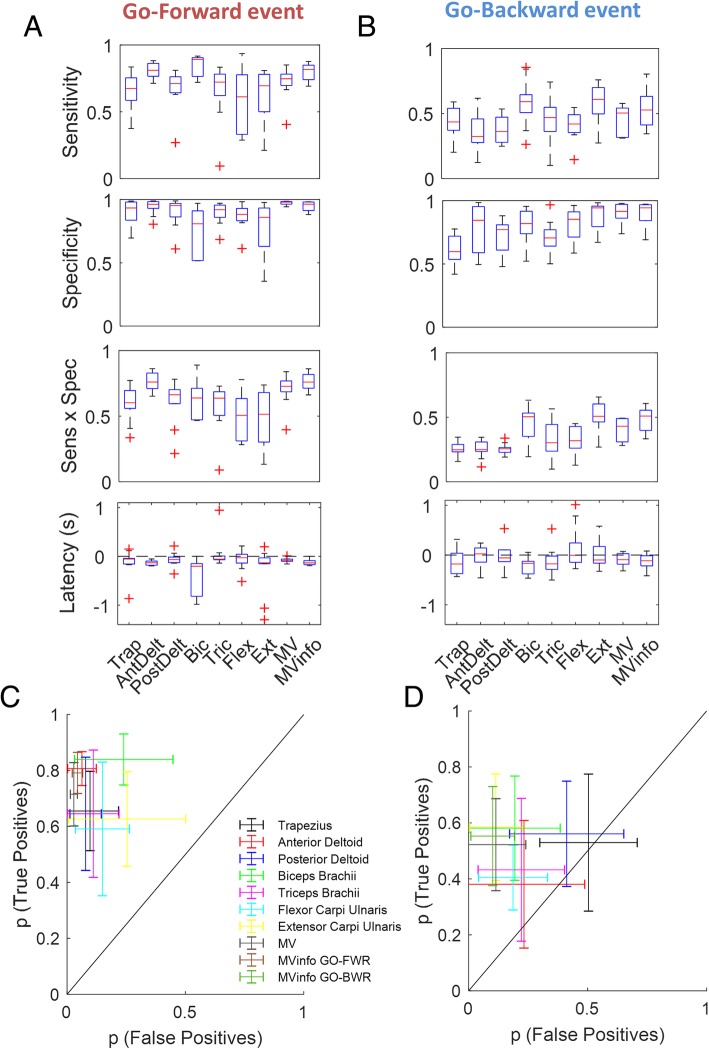
Performance metrics of the detectors for *Go-forward* (**a**) and *Go-backward* (**b**). Detection results in true positive- false positive rate for *Go-forward* (**c**) and *Go-backward* (**d**): the mean value and standard deviation bars are reported for each detector

Figure 5a and b show, for each of the two events, the performance of the three types of detector that were tested, in terms of sensitivity, specificity and latency. Regarding *Go-forward* onset detection, Type 1 detectors receiving as input single EMG signals from Anterior Deltoid and Biceps show higher sensitivity with respect to the other muscles. The median sensitivity (and interquartile range) is equal to 81.1% (76.6–86.24%) and 89.3% (90.5–76.5%) respectively. Nevertheless, whereas Anterior Deltoid detector has the highest specificity among Type 1 detectors (96.2% (93.0–98.3%)), Biceps detector exhibit the lowest specificity, equal to 80.8% (51.8–91.0%). Median latency values range from − 0.202 s (Biceps) to − 0.029 s (Flexor Carpi Ulnaris). The Type 2 detector (Majority Voting) exhibits the highest specificity (median value of 97.9% (98.4–96.3%); however, it performs worse than other Type 1 detectors in terms of sensitivity (74.7% (69.7–78.2%)). Median latency is equal to − 0.088 s (− 0.101 – − 0.057 s). By choosing only the most informative muscles as input to the Type 2 detector, the sensitivity increases to 81.7% (74.0–84.9%), while specificity and latency slightly decrease to 96.3% (91.3–98.6%) and − 0.133 s (− 0.168 – − 0.087 s) respectively.

With reference to *Go-backward*, Extensor Carpi Ulnaris and Biceps Type 1 detectors exhibit the highest sensitivity (median values of 60.9% (49.7–69.9%) and 59.1% (50.7–64.5%) respectively) with respect to the other type of detectors. Extensor Carpi Ulnaris Type 1 detector exhibits the highest specificity as well, with a median value of 94.3% (79.5–95.7%), and median latency equal to − 0.099 s (− 0.163–0.173 s). All other Type 1 have a sensitivity below 50%. Type 2 detector (Majority Voting an all muscles) resulted to have poor sensitivity for *Go-backward* (median value of 50.4% (31.7–54.2%)) and specificity equal to 91.6% (86.2–97.1%). Median latency is equal to − 0.093 s (− 0.184–0.028 s). When only the most informative muscles are selected for the Type 2 detector, sensitivity and specificity increase to 52.7% (41.2–63.2%) and 94.3% (84.2–96.7%). Latency decreases to − 0.115 s (− 0.217 – − 0.014 s). Table 2 summarizes the best performances for each parameter and for each detector.

**Table 2:** Performances of the three types of detector for the two events. For Type 1 detectors, performances of the source with the best performances (Sens.*Spec.) are reported, which is Anterior Deltoid (AD) for *Go-forward* and Biceps Brachii (BB) for *Go-backward*

	Go-forward			Go-backward		
Detector	Type 1 (AD)	Type 2	Type 2-info	Type 1 (EXT)	Type 2	Type 2-info
Sens. (%)	81.1 (76.5–90.5)	74.7 (69.7–78.2)	81.6 (76.2–84.0)	60.9 (49.7–69.9)	50.4 (31.7–54.2)	52.7 (41.2–63.2)
Spec. (%)	96.2 (93.0–98.3)	97.9 (96.3–98.4)	96.3 (91.3–98.0)	94.3 (79.5–95.7)	91.6 (86.2–97.1)	94.3 (84.2–96.7)
Latency (s)	−0.130 (− 0.181 – − 0.096)	−0.088 (− 0.101–0.057)	−0.134 (− 0.169 – − 0.088)	−0.099 (− 0.163–0.173)	−0.093 (− 0.184–0.028)	−0.115 (− 0.217 – − 0.014)

Figure 5c and d show, for the two events respectively, the true positive- vs false positive- rate calculated over all the trials performed by each subject and for each detector (mean values and standard deviations are reported for both measures). Type 2 detector exhibits the highest mean ratio for *Go-forward*, equal to 23.7 (0.71/0.03), which slightly decreases to 19.7 (0.79/0.04) when only the most informative muscles are selected. Among Type 1 detectors, Anterior Deltoid has the highest mean ratio, equal to 13.5 (0.81/0.06). As of *Go-backward*, Extensor Carpi Ulnaris and Biceps Brachii exhibit the highest ratios among Type 1 detectors, equal to 5.3 (0.58/0.11) and 3.1 (0.58/0.19) respectively. Type 2 Info-based detector performs slightly better than the others, having a ratio equal to 5.5 (0.55/0.10).

#### Computational load

Results from the simulation on the real-time controller revealed that the maximum iteration duration for event detection is lower than 2 ms. The maximum value of iteration duration allowed by the controller for real-time operation is 10 ms.

## Discussion

Classification methods based on GMM have been implemented for the myoelectric control of assistive devices such as prostheses or robotic arms [17, 32]. In our work, a GMM-based algorithm has been implemented and information theory was used to identify the best set of features able to detect the onset of upper-limb muscular activation, with the final goal of controlling a robotic exoskeleton for assistive tasks. Among the 14 time-domain features selected, a first screening was conducted based on their information content with respect to white noise. The smallest number of features which maximize synergistic effects and minimize redundancy effects is selected, in order to: i) reduce the probability that two different features will share the same information about rest/movement states (redundancy); ii) reduce the probability that the information content of the features alone is higher with respect to the information of the coupled features (synergy). Clearly, selecting the smallest number of features would be recommended for online applications, in order to reduce computational load and achieve faster detection without degradation of the performances. Among the ones we selected, IAV and WL have also been exploited to extract useful information about muscles activation [19, 32–34]. Although some of the features analyzed in this study have a similar formulation (such as IAV, MAV, MMAV1, MMAV2), we did not make a biased selection of features based on a priori knowledge of their definition or their similarities and used information theory to rule out redundant and non synergistic features. Indeed, previous works have shown that similar EMG features or their combination can yield significantly different results [35, 36]. We compared performance metrics of both *Go-forward* and *Go-backward* using all 14 features with the results obtained by the optimal subset. Only *Go-backward* sensitivity was slightly but significantly higher when using all features (median difference 0.0086; Wilcoxon sign rank test, *p* < 0.001). All the other performance indices were not significantly different (Wilcoxon sign rank test, *p* > 0.05), showing that usage of information theory to reduce the number of features did not affect overall detection performance, allowing in parallel a reduction of the total computational load of the detection algorithms (approx. 80% reduction).

The importance of the information content on the accuracy of onset detection was confirmed by the in-depth analysis on the four chosen features. In particular, the positive correlation between the detector performance (which takes into account both sensitivity and specificity) of the Type 1 detectors and the information content of the extracted features suggests that higher information content can be associated to better event recognition. Thus, an a priori analysis based on the breakdown of information can be useful to identify which features would be more effective for accurate detection of the movement onset. Indeed, among Type 1 detectors, features extracted from Anterior and Posterior Deltoid carry the highest information and have the highest combination of sensitivity and specificity for *Go-forward*. Similarly, Biceps and Extensor Carpi Ulnaris have the highest information for *Go-backward*. A similar trend between information content and performances can be observed by comparing Figs. 4 and 5A-B (Sens. x Spec. panel). The information content of the features for the Type 1 detectors of *Go-Backward* reflects on the worse performance of its detection with respect to *Go-forward*, for which sensitivity, specificity and information content are overall higher. Indeed, the true positive- vs false-positive rate ratio is always higher than 1 for *Go-forward* for all detectors, whereas for *Go-backward* the distributions are widely spread, and some detectors exhibit a ratio lower than 1 or close to it. The high information content for *Go-forward* carried by most of the Type 1 detectors for *Go-forward* suggests that a particular combination of them through a majority voting could provide accurate detection as well. Taking into account contributions from all muscles is disadvantageous for both events detection, because of the scarce information associated to this event by some of the single detectors. Conversely, given the positive correlation between information and detector performance, the selection of the most informative muscles as input to the Type 2 detector gives acceptable performances in terms of sensitivity and specificity, while reducing the computational load in the training and testing phase with respect to considering all muscles.

The two events have been chosen in order to simulate different conditions of real-life scenario, which can vary extensively according to the environment and the subjects’ residual motion capabilities [37]. As an example, *Go-forward* is typical in situations where the user wants to initiate a new task or activity and the robot can modulate the level of assistance, up to providing passive mobilization, in the worst scenarios. On the contrary, *Go-backward* reflects situations in which sequential movements must be performed (e.g. a complete functional tasks composed of different sub-actions) and significant changes in the background noise level of EMG signals can be encountered in relatively short time.

Although the highest sensitivity for *Go-forward* event is higher than 80% when the proper detector is used, sensitivity for *Go-backward* detection is around 60% in the best case. The differences in the recognition of the two events could be due to the particular conditions of the experiments. Indeed, whereas *Go-forward* corresponds to a transition from rest to movement state when the user is completely relaxed (the exoskeleton is in transparent mode), the initial condition for Go-backward was with the arm stretched toward the target and the exoskeleton controlled in position mode, restraining any movement of the user. This condition did not allow subjects to relax their muscles before activating the *Go-backward* transition. In addition, the time interval for which the phase signal is 1 (i.e. movement state) is shorter for *Go-backward* than for *Go-forward* and it is dependent on the torque threshold chosen for the activation. This has two main implications: first, the sequential algorithm that adaptively modifies GMM parameters works on a shorter time window, reducing the accuracy in the calculation of the time-varying threshold to discriminate rest/movement states; furthermore, by selecting a higher torque threshold for *Go-Backward* initiation, a volitional muscular activity could have been better discriminated during the training phase. A low torque threshold has been selected for the experiments in order to reduce fatigue effects on the subjects.

The low sensitivity of the *Go-backward* detection represents the main limitation of the proposed method, which would make it difficult for the user to retract from the reaching position in real-time applications. Before the *Go-backward* event, the arm was completely extended toward the target, and held steady by the exoskeleton. In such position, the arm muscles exhibited a residual activation, which was non-optimal for discriminating between rest and movement states. In fact, although the subjects were instructed to keep their muscles as relaxed as possible, such activation increased the “background noise” on the EMG signals, leading to poor detection performance even with the best parameters and the optimal window length. A possible solution to address this problem would require a modification of the experimental protocol with respect to the rest positions for the Go-backward event. For example, a more comfortable posture with the arm not completely extended toward the target could help the subjects to keep their muscles relaxed. As a result, the information content carried by the EMG signals about the event would increase, thus improving detection performance.

The results about latency are comparable to other systems in the state of the art. For example, the multimodal control system in [9] is capable to predict movement onset via EMG analysis with a prediction time of 0.061 s, which was reduced to a value of 0.057 s when EMG and EEG signals were combined in a hybrid fashion. The GMM method presented in this paper has the advantage of using only EMG signals to predict the onset of the movement from 0.088 s up to 0.134 s (Table 2, *Go-forward*), reducing the complexity of the system while maintaining acceptable performances in terms of sensitivity and specificity, higher than 80 and 96% respectively for *Go-forward*. Earlier predictions have also been recorded, up to 0.202 s (median value) for Biceps Type 1 detector in *Go-forward*, but with poor overall performances. It is worth noticing that here latency is defined as the time delay between muscular activation onset and kinematic onset, rather than the delay between the algorithm detection and actual muscle activation. Thus, negative values of latency are preferred in order to design a control strategy able to react promptly to the user’s intention. In this case, by taking into account the contribution of a specific muscle or a sub-set of muscles to a certain movement, it would be possible to trigger the robot assistance before having a substantial modification of the kinematic metrics, which would be strenuous for users with highly-reduced mobility of their upper arm.

When used in conjunction with the upper-limb exoskeleton, smart algorithms can be combined in order to reduce the effect of false activations. As an example, a robot-assisted full functional task can be implemented by means of a finite-state machine to split the main task in different sub-actions. Then, event detection can be triggered only when the proper state is activated. Similar approaches have been pursued, but using different interfaces for detecting the user’s intention to move [10, 38]. Another study on healthy subjects showed that combining EMG data with kinematic data from the exoskeleton can improve the performance of classification of the movement direction, with respect to using EMG signals alone [39]. A hybrid approach exploiting kinetic data from the exoskeleton could allow to deal with pathological sEMG as well, as of post-stroke subjects exhibiting arm spasticity. In this case, an involuntary muscle contraction due to a spasm could be detected by the onboard torque sensors of the exoskeleton [40], and the event detection would be neglected. Future studies need to be conducted in order to evaluate the feasibility of such approach for online applications.

## Conclusions

In this paper, we presented an algorithm for the detection of the user’s intention to move based on the onset of muscular activity. We found that information theory represents a powerful tool to predict which features could be more representative for an accurate and robust detection of a desired event. For offline analysis, kinematic data of the upper-limb exoskeleton have been exploited to discriminate two different events, reproducing possible scenarios of daily-life activities for people with reduced mobility of their upper arm. The performances of different detectors have been analyzed, showing that information from single muscles or a combination of them can be equivalently effective depending on the kinematic event that is considered. Although the performance of the algorithm has been tested offline, its applicability to real scenario has been discussed. The capability to predict the onset of muscular activity before the kinematic event takes place, the accurate detection and the low computational load make the proposed algorithm promising for the control of upper-limb exoskeletons in online applications. The final goal would be aiding people with severe arm disabilities in performing assisted functional tasks. Clearly, additional test will be required in order to assess the performance of the algorithm when non-physiological muscle activation patterns are used.

## Appendix

### Feature Definitions

According to [26], the features considered in this study are defined as the following:

*Integrated Absolute Value (IAV):* It is calculated as the summation of the absolute values of the signal amplitude in the window frame,

$$ IAV=\sum \limits_{n=1}^N\left|{x}_n\right| $$

where *N* is the number of samples of the sliding window (*N* = 100).

*Mean Absolute Value (MAV)*: It is evaluated by taking the average of each signal,

$$ MAV=\frac{1}{N}\sum \limits_{n=1}^N\left|{x}_n\right| $$

*Modified Mean Absolute Value 1 (MMAV1)*: It is an extension of MAV which uses a weighting window function,

$$ MMAV1=\frac{1}{N}\sum \limits_{n=1}^N{w}_n\left|{x}_n\right| $$

$$ {w}_n=\left\{\begin{array}{cc}1&\ if\ 0.25N\le n\le 0.75N\\ {}\ 0.5& otherwise\end{array}\right.. $$

*Modified Mean Absolute Value 2 (MMAV2)*: It is similar to MMAV1, with a different weighting function,

$$ MMAV2=\frac{1}{N}\sum \limits_{n=1}^N{\widehat{w}}_n\left|{x}_n\right| $$

$$ {\widehat{w}}_n=\left\{\begin{array}{cc}1& if\ 0.25N\le n\le 0.75N\\ {}4n/N& if\ 0.25N>n\\ {}4\left(n-N\right)/N& if\ 0.75N<n\end{array}\right. $$

*Simple Square Integral (SSI):* It is the energy of the signal,

$$ SSI=\sum \limits_{n=1}^N{x_n}^2 $$

*Variance (VAR)*: It is the variance of the signal,

$$ VAR=\frac{1}{N-1}\ \sum \limits_{n=1}^N{\left({x}_n-\mu \right)}^2 $$

where *μ* is the average value of the signal.

*Root Mean Square (RMS)*: It is the root of the mean squared signal,

$$ RMS=\sqrt{\frac{1}{N}\ \sum \limits_{n=1}^N{x_n}^2} $$

*Waveform Length (WL)*: It is the cumulative length of the waveform,

$$ WL=\sum \limits_{n=1}^{N-1}\left|{x}_{n+1}-{x}_n\right| $$

*Willison Amplitude (WAMP):* It is the number of times that the difference between two adjacent amplitude values exceeds a predefined threshold,

$$ WAMP=\sum \limits_{n=1}^{N-1}f\left(\left|{x}_{n+1}-{x}_n\right|\right) $$

$$ f(x)=\left\{\begin{array}{cc}1&\ if\ x\ge threshold\\ {}0& otherwise\end{array}\right. $$

*Slope Sign Change (SSC):* It is the number of changes between positive and negative slope; it is calculated by using a threshold function to minimize the influence of noise in the signal,

$$ SSC=\sum \limits_{n=2}^{N-1}f\left[\left({x}_n-{x}_{n-1}\right)\left({x}_n-{x}_{n+1}\right)\right] $$

$$ f(x)=\left\{\begin{array}{cc}1&\ if\ x\ge threshold\\ {}0& otherwise\end{array}\right. $$

*Zero-Crossing (ZC):* It is the number of times that the amplitude of the signal crosses the zero value,

$$ ZC=\sum \limits_{n=1}^{N-1}\left[\mathit{\operatorname{sign}}\left({x}_n\times {x}_{n+1}\right)\cap \left|{x}_n-{x}_{n+1}\right|\ge threshold\right] $$

$$ \mathit{\operatorname{sign}}(x)=\left\{\begin{array}{cc}1&\ if\ x\ge threshold\\ {}0& otherwise\end{array}\right. $$

*Logarithm (LOG):* It is the mean of the common logarithm of the absolute value of the signal,

$$ LOG=\frac{1}{N}\sum \limits_{n=1}^N{\log}_{10}\left(\left|{x}_n\right|\right) $$

*Skewness (SKEW):* It is the third standardized moment, defined as:

$$ SKEW=\frac{\frac{1}{N}\ \sum \limits_{n=1}^N{\left({x}_n-\mu \right)}^3}{{\left(\frac{1}{N-1}\ \sum \limits_{n=1}^N{\left({x}_n-\mu \right)}^2\right)}^{3/2}} $$

where *μ* is the average value of the signal.

*Kurtosis (KURT):* It is the fourth standardized moment, defined as:

$$ KURT=\frac{\frac{1}{N}\ \sum \limits_{n=1}^N{\left({x}_n-\mu \right)}^4}{{\left(\frac{1}{N-1}\ \sum \limits_{n=1}^N{\left({x}_n-\mu \right)}^2\right)}^2} $$

## Abbreviations

AD: Anterior deltoid
BMI: Brain-machine interface
cHRI: Cognitive human-robot interface
DMA: Direct Memory Access
DOF: Degree of freedom
EEG: Electroencephalography
eFE: Elbow flexion-extension
EM: Expectation maximization
EOG: Electrooculography
EXT: Extensor Carpi Ulnaris
FIFO: First-In-First-Out
FN: False negative
FP: False positive
FPGA: Field-programmable gate array
GMM: Gaussian mixture model
GO-BWR: Go-backward event
GO-FWR: Go-forward event
GUI: Graphical user interface
IAV: Integrated absolute value
KW: Kruskal-Wallis
LOG: Logarithm
MAV: Mean absolute value
MLE: Maximum likelihood estimation
MMAV1: Modified mean absolute value 1
NESM: NeuroExos Shoulder-Elbow Module
NI: National instrument
PID: Proportional-integrative-derivative
RMS: Root mean square
sAA: Shoulder abduction-adduction
SEA: Series-elastic actuator
sEMG: Surface electromyography
SENS.: Sensitivity
sFE: Shoulder flexion-extension
sIE: shoulder internal-external rotation
SPEC.: Specificity
SSI: Simple square integral
TN: True negative
TP: True positive
VAR: Variance
WL: Waveform length
